# The Effect of Caffeine on Various Forms of Synaptic Plasticity in the CA1 Region of Mouse Hippocampal Slices

**DOI:** 10.3390/biom16050740

**Published:** 2026-05-19

**Authors:** Margarita A. Novikova, Irina A. Korneeva, Rodion V. Kondratenko, Georgii M. Nikolaev, Olga A. Averina, Irina N. Sharonova, Alexander V. Latanov

**Affiliations:** 1 Faculty of Biology, Lomonosov Moscow State University, 119234 Moscow, Russia; irina.korneeva1212@gmail.com (I.A.K.); gnikolaev@neurobiology.ru (G.M.N.); latanov.msu@gmail.com (A.V.L.); 2 A.N. Belozersky Institute of Physico-Chemical Biology, Lomonosov Moscow State University, 11999 Moscow, Russia; averina.olga.msu@gmail.com; 3 Functional Synaptology Laboratory, Brain Science Institute, Russian Center of Neurology and Neurosciences, 125367 Moscow, Russia; kondrat_r@mail.ru (R.V.K.); sharonova.irina@gmail.com (I.N.S.); 4 Research Institute for Brain Development and Peak Performance, Peoples’ Friendship University of Russia, 117198 Moscow, Russia

**Keywords:** caffeine, LTP, E-S potentiation, hippocampal CA1 area, hippocampal slices, input–output relationships, paired-pulse ratio

## Abstract

Caffeine is the most widely used psychoactive compound. In the brain, caffeine acts as a competitive, non-selective adenosine receptor antagonist of A_1_ and A_2A_, both known to modulate long-term potentiation (LTP), the cellular basis of learning and memory. But the effects of caffeine on synaptic function and plasticity cannot be reduced to a single inhibitory or facilitatory action. In the CA1 area of the hippocampus, low-micromolar caffeine has been reported to attenuate LTP, yet it remains unclear whether this action extends equally to other plasticity-related responses, including EPSP–spike coupling and paired-pulse responses. Here, we studied the effect of 30 μM caffeine on the field excitatory postsynaptic potentials (fEPSPs) and LTP evoked by Schaffer collateral stimulation in the CA1 region in mouse hippocampal slices. We compared theta-burst-induced long-term fEPSP potentiation, EPSP–spike (E-S) potentiation, input–output relationships, and paired-pulse responses after short (three burst-TBS3) and long (ten burst-TBS10) theta-burst stimulation. Caffeine attenuated long-term fEPSP potentiation induced by the longer theta-burst protocol and reduced the accompanying increase in population spike amplitude. In contrast, E-S potentiation induced by the shorter theta-burst protocol was preserved under caffeine exposure. Input–output analysis further showed that caffeine prevented the increase in population spike amplitude accompanying the development of long-term fEPSP potentiation, but did not prevent the population spike response changes associated with E-S potentiation. Caffeine also reduced paired-pulse deviations from 100%, most clearly for population spike amplitude, and this effect persisted after both the theta-burst protocols. Thus, 30 μM caffeine did not simply suppress CA1 plasticity-related responses, but distinguished TBS10-induced synaptic fEPSP potentiation from TBS3-induced EPSP–spike potentiation. These findings identify EPSP–spike coupling as a caffeine-preserved CA1 plasticity-related response and provide a basis for future receptor-selective and behavioral testing.

## 1. Introduction

Caffeine is the most widely consumed psychoactive compound and is present in coffee, tea, energy drinks, food products, and medications [1,2]. After ingestion, caffeine is rapidly absorbed, distributed through biological tissues, and crosses the blood–brain barrier [1,2,3]. At low micromolar CNS concentrations reported after moderate caffeine intake, its main CNS action is generally attributed to non-selective antagonism of adenosine receptors [1,2]. Phosphodiesterase inhibition and intracellular Ca^2+^ mobilization require substantially higher caffeine concentrations and are therefore not considered primary mechanisms of low-micromolar caffeine action [1,2].

Adenosine acts through four P1 receptor subtypes, A_1_, A_2A_, A_2B_ and A_3_, which differ in affinity, intracellular coupling, and tissue distribution [4,5]. Basal extracellular adenosine strongly recruits high-affinity A_1_ receptors, whereas A_2A_ receptor engagement depends more on local adenosine availability, receptor localization, and activity state [4,5]. A_1_ receptors are mainly coupled to G_i/o_ proteins, whereas A_2A_ receptors are mainly coupled to G_s/olf_ proteins [4,5]. In the nervous system, adenosine regulates basal synaptic transmission, neuronal excitability, and the threshold for activity-dependent plasticity mainly through A_1_ and A_2A_ receptor-dependent mechanisms [5]. Adenosine can also modulate inhibitory transmission, which is relevant as a circuit-level caveat when interpreting field responses [6].

The hippocampal CA3-CA1 pathway has been widely used to study adenosine-dependent modulation of synaptic transmission and plasticity [5,7,8]. In Schaffer collateral-CA1 synapses, previous slice experiments showed that low-micromolar caffeine can modify basal synaptic transmission, paired-pulse facilitation (PPF) and long-term potentiation (LTP) [7,8]. In receptor-selective experiments, caffeine-sensitive facilitation of synaptic transmission was associated with antagonism of presynaptic A_1_ receptors, whereas caffeine-sensitive reduction in LTP was attributed to A_2A_ receptor blockade [8]. Chronic caffeine exposure also modifies synaptic function, metabolism and adenosine modulation in a brain region-dependent manner [9]. Together, these studies identify basal synaptic transmission, paired-pulse facilitation (PPF), and LTP as caffeine-sensitive readouts in hippocampal slice preparations [7,8].

Long-term potentiation is a major experimental model of activity-dependent strengthening of excitatory synaptic transmission [10,11]. Its induction and expression depend on synapse type, circuit organization, developmental stage, and stimulation protocol [12,13]. In extracellular recordings from the CA1 region, afferent stimulation evokes both a field excitatory postsynaptic potential (fEPSP) in the dendritic layer and a population spike in the pyramidal cell layer [10,14]. Early hippocampal slice experiments showed that potentiation can involve changes in the fEPSP, population spike amplitude, firing probability, and spike latency [14].

In CA1 field recordings, high-frequency stimulation can change not only the fEPSP and population spike, but also the relationship between synaptic input and spike output [15,16]. This component of potentiation is referred to as EPSP–spike, or E-S, potentiation and denotes a long-lasting increase in population spike output relative to the excitatory synaptic response [15,16]. Because the synaptic component in field recordings is commonly quantified by EPSP/fEPSP slope and the spike output by population spike amplitude, E-S potentiation is assessed from the relationship between these two parameters [16,17]. A shift in this relationship indicates that a given EPSP slope evokes a larger population spike, or that a given population spike is reached at a smaller EPSP slope [16,17,18]. This measure reflects EPSP-to-spike coupling rather than synaptic strength alone [15,19].

E-S potentiation can be operationally separated from synaptic LTP in extracellular recordings [15,17,20]. Short trains of theta-frequency stimulation in CA1 can induce persistent population spike potentiation without lasting fEPSP slope potentiation, indicating that synaptic potentiation and EPSP–spike coupling can be modified separately [20]. Recent studies continue to use E-S potentiation as an additional readout of hippocampal output modulation, including conditions in which population spike amplitude increases without significant fEPSP potentiation [21,22]. Therefore, fEPSP potentiation and E-S potentiation represent related but separable CA1 plasticity-related electrophysiological responses. This distinction provides the basis for testing whether low-micromolar caffeine uniformly affects selected CA1 plasticity-related electrophysiological readouts or differentially modifies synaptic potentiation and EPSP–spike coupling.

In addition to long-lasting potentiation, caffeine-sensitive modulation may also appear in short-term responses to repeated afferent activation. Paired-pulse responses, obtained by comparing the response to a second stimulus with the response to the first stimulus, provide a standard protocol for assessing responses to two consecutive afferent stimuli in hippocampal CA1 [23,24]. Although PPF is often considered in relation to transmitter release probability, paired-pulse field responses combine synaptic and circuit-level components, including inhibitory conductances [24,25,26,27,28]. In this context, paired-pulse responses complement fEPSP-based LTP and E-S potentiation as a short-term readout of CA1 response modulation.

The present study compared the sensitivity of selected CA1 plasticity-related electrophysiological responses to 30 μM caffeine. We analyzed long-term fEPSP potentiation, E-S potentiation, input–output relationships, and paired-pulse responses in mouse hippocampal slices. This approach tested whether 30 μM caffeine similarly affects these responses or differentially affects synaptic potentiation, EPSP–spike coupling, population spike output, and responses to paired stimulation.

## 2. Materials and Methods

### 2.1. Animals

Specific pathogen-free male C57BL/6×CBA F1 hybrid mice aged 6–10 weeks were used in this study. The animals were obtained from the Transgenesis Laboratory of the Department of Functional Genomics, Lomonosov Moscow State University. Before transportation to the vivarium, mice were maintained at the supplier facility in standard individually ventilated cages. Only male mice were used because the present study was designed as an ex vivo electrophysiological analysis of caffeine effects on defined CA1 plasticity-related responses, without sex and estrous-cycle stage as additional experimental variables. This design was chosen in view of previous slice electrophysiology studies showing that estrous-cycle stage can affect CA1 LTP magnitude [29] and that sex differences in hippocampal LTP can depend on the activated pathway and stimulation protocol [30].

Immediately after transportation to the vivarium, the animals were placed in quarantine in a separate room for 10 days to minimize possible stress-related effects of transportation. After quarantine, the animals were transferred to the main vivarium room. Mice were maintained at controlled temperature (23 ± 2 °C) under a fixed 12 h light/dark cycle, with free access to food and water. In the vivarium, animals were housed in standard polycarbonate cages for small rodents with a floor area of 335 cm^2^. Small cylinders or tunnels made of food-grade cardboard were used for environmental enrichment.

All experimental procedures were approved by the Institutional Ethics Committee of the Research Center of Neurology (Protocol No. 10-8/21, 17 November 2021) and were performed in accordance with Russian national regulations and the European Community directive for the use of experimental animals.

### 2.2. Hippocampal Slice Preparation and Extracellular Recordings

Mice were euthanized by cervical dislocation followed by decapitation. The brain was rapidly removed and placed in ice-cold artificial cerebrospinal fluid (ACSF) continuously equilibrated with carbogen (95% O_2_, 5% CO_2_). ACSF contained, in mM: 124.0 NaCl, 4.4 KCl, 1.0 Na_2_HPO_4_, 25.0 NaHCO_3_, 2.0 CaCl_2_, 1.0 MgCl_2_, and 10.0 glucose.

Acute transverse hippocampal slices were prepared from the dorsal hippocampus. Slices were approximately 450 μm thick and were cut using a custom-built vibroknife. Usually, 3–6 slices were obtained from one brain. Slices were transferred to an incubation chamber using a clipped Pasteur pipette. The time from euthanasia to transfer of hippocampal slices into the incubation chamber did not exceed 3 min.

Slices were allowed to recover for at least 1 h before electrophysiological recording. During recovery, all slices were maintained in caffeine-free ACSF at room temperature and continuously equilibrated with carbogen. Recordings were performed in a submerged recording chamber continuously perfused with ACSF delivered at a flow rate of 5–8 mL/min; ACSF was continuously equilibrated with carbogen. The temperature in the recording chamber was maintained at approximately 30 °C.

The recording setup contained a single recording chamber and allowed recording from only one slice at a time. Therefore, one slice from each animal was selected for recording and subsequent analysis. When several dorsal hippocampal slices were available from the same animal, the slice for recording was selected before electrophysiological testing based only on visual integrity of the hippocampal formation and the absence of obvious mechanical damage. This approach also reduced the variability associated with different storage times of slices before recording. Since only one slice from each animal was included in the final analysis, n corresponds to both the number of slices/recordings and the number of animals.

Caffeine was not present during slice recovery. In caffeine-treated groups, 30 μM caffeine was added only to the ACSF perfusing the recording chamber. After electrode positioning, slices were allowed to stabilize for 15 min before baseline acquisition. In caffeine-treated groups, this stabilization period was performed in caffeine-containing ACSF, and caffeine remained present throughout the subsequent recording session. Control slices were perfused with caffeine-free ACSF in the recording chamber for the same period and throughout the experiment.

A bipolar Teflon-insulated tungsten stimulation electrode was placed in the stratum radiatum at the CA1-CA2 border to activate Schaffer collateral inputs. Two glass capillary recording electrodes filled with 1.5 M NaCl and containing AgCl wires were used for extracellular recordings. One recording electrode was placed in the CA1 stratum radiatum to record field excitatory postsynaptic potentials (fEPSPs), and the second recording electrode was placed in the CA1 stratum pyramidale to record population spikes.

Stimulation protocols and data acquisition were controlled using WinWCP 5.4.2 software (University of Strathclyde, UK). Signals were recorded using 1 Hz high-pass and 1 kHz low-pass filtering, 1000-fold amplification, and a sampling rate of 20 kHz. Quantitative analysis was performed in Clampfit 10.7 (Axon Instruments, Union City, CA, USA).

For each evoked response, the fEPSP slope and population spike amplitude were measured. These parameters were used as extracellular readouts of synaptic input and population spike output, respectively [31]. The fEPSP slope was calculated in Clampfit after manual placement of two cursors on the most linear segment of the rising phase of the fEPSP. Population spike amplitude was calculated in Clampfit as the voltage difference between the minimum and maximum response values within a manually defined analysis window containing the population spike.

After the 15 min stabilization period, baseline responses were evoked by 0.1 ms stimuli delivered once every 30 s. Stimulation intensity was adjusted to evoke 60–70% of the maximal population spike amplitude, estimated from an input–output curve or a maximal-response test before baseline recording. Recordings were included in the analysis only if they showed a stable baseline response, clearly distinguishable fEPSP and population spike components, and no progressive response drift. Recordings that failed to achieve response stability within 10 min of baseline stimulation were excluded from the analysis.

### 2.3. Experimental Design and Groups

Electrophysiological experiments were organized according to the stimulation protocol and the measured response type. Control slices were recorded in caffeine-free ACSF, whereas caffeine-treated slices were exposed to 30 μM caffeine in the ACSF perfusing the recording chamber, as described above.

Long-term responses induced by theta-burst stimulation (TBS) were analyzed in four groups. TBS10-induced responses were recorded in caffeine-free ACSF (control TBS10, n = 13) and during acute exposure to 30 μM caffeine (caffeine TBS10, n = 14). TBS3-induced responses were recorded in caffeine-free ACSF (control TBS3, n = 16) and during acute exposure to 30 μM caffeine (caffeine TBS3, n = 17).

Input–output relationships and paired-pulse responses were analyzed using the same six-group design. These groups included slices recorded without TBS in caffeine-free ACSF (control without TBS), slices recorded without TBS during acute exposure to 30 μM caffeine (caffeine without TBS), slices subjected to TBS10 in caffeine-free ACSF (control TBS10), slices subjected to TBS10 during acute caffeine exposure (caffeine TBS10), slices subjected to TBS3 in caffeine-free ACSF (control TBS3), and slices subjected to TBS3 during acute caffeine exposure (caffeine TBS3); n = 7 in each group.

### 2.4. Theta-Burst Stimulation Protocols and Quantification of Long-Term Responses

After a stable 10 min baseline recording, theta-burst stimulation (TBS) was delivered once at the same stimulation intensity as baseline test stimulation. Baseline values were calculated by averaging all responses recorded during the 10 min period immediately preceding TBS. All subsequent fEPSP slope and population spike amplitude values were normalized to the corresponding baseline values and expressed as percentages of baseline.

Two TBS protocols were used. The TBS10 protocol consisted of 10 bursts, each containing 4 pulses delivered at 100 Hz, with an inter-burst interval of 160 ms. The TBS3 protocol consisted of 3 bursts with the same intra-burst structure and inter-burst interval. TBS10 was used to induce long-term potentiation of the fEPSP slope, whereas TBS3 was used to induce EPSP–spike potentiation.

For TBS10 experiments, long-term responses were quantified as the mean normalized fEPSP slope and population spike amplitude during 35–40 min after TBS10. For TBS3 experiments, long-term responses were quantified using the same 35–40 min post-TBS interval.

EPSP–spike (E-S) potentiation was defined as a long-lasting increase in population spike amplitude relative to fEPSP slope, reflecting a change in the relationship between the evoked synaptic field response and population spike output. In the present experiments, TBS3-induced E-S potentiation was assessed using the mean normalized fEPSP slope and population spike amplitude during 35–40 min after TBS3. The TBS3 protocol was operationally considered to induce E-S potentiation when population spike amplitude increased without a comparable increase in fEPSP slope.

### 2.5. Input–Output Relationships

Input–output relationships were used to assess stimulation-dependent changes in CA1 excitability-related field responses. Stimulation intensity was increased from the threshold intensity (×) to 7.5-fold threshold intensity (7.5×). The lowest intensity was defined as the threshold for evoking a visually detectable fEPSP and was taken as 1×. The remaining intensities were set as multiples of this threshold: 1.3×, 1.8×, 2.4×, 3.2×, 4.2×, 5.6×, and 7.5×. This threshold-based logarithmic scale was adapted from previous slice electrophysiology studies using input–output protocols to cover a broad range of stimulation-dependent changes in evoked hippocampal field responses [32].

At each stimulation intensity, two evoked responses were recorded and averaged for each slice. Stimuli were 0.1 ms in duration. The interval between repeated stimulations at the same intensity was 30 s.

For each stimulation intensity, fEPSP slope and population spike amplitude were measured as described above. These curves were used to compare stimulation-dependent recruitment of the fEPSP and population spike components of the evoked CA1 response. This approach is consistent with the use of input–output profiles to describe changes in the output of a neuronal population across a range of input intensities [33].

To reduce the influence of between-slice variability in absolute field potential amplitude, including variability related to slice geometry and electrode positioning, responses were expressed as a percentage of the mean response recorded during the corresponding 10 min baseline period at the baseline test stimulation intensity. In groups without TBS, this baseline period was recorded immediately after the input–output protocol. In the TBS10 and TBS3 groups, the pre-TBS baseline period was used as the reference for normalization.

### 2.6. Paired-Pulse Responses

Paired-pulse stimulation was used to assess evoked CA1 field responses to two consecutive afferent stimuli at predefined interstimulus intervals. In groups without TBS, this protocol followed the 10 min baseline recording; in TBS10 and TBS3 groups, it followed the 40 min post-TBS recording period and input–output series. For paired-pulse testing, stimulation intensity was returned to the baseline test intensity used for the corresponding slice, i.e., the intensity adjusted before baseline recording to evoke 60–70% of the maximal population spike amplitude. Paired-pulse stimulation is widely used in CA1 recordings to assess responses to repeated afferent activation, including Schaffer collateral-evoked excitatory responses, inhibitory recruitment, and population spike output [34].

Paired stimulation was delivered at eight interstimulus intervals: 15, 30, 50, 80, 100, 150, 250, and 400 ms. Each stimulus was 0.1 ms in duration. For each interstimulus interval, three paired responses were recorded, with paired stimulation delivered once every 30 s. The three responses obtained at each interstimulus interval were averaged for each slice before statistical analysis.

The paired-pulse ratio (PPR) was calculated for each interstimulus interval as the second-to-first response ratio multiplied by 100% (P2/P1 × 100%), consistent with paired-pulse analysis in hippocampal CA1 recordings [23]. This calculation was performed separately for the two recorded parameters: fEPSP2 slope/fEPSP1 slope × 100% for fEPSPs and PS2 amplitude/PS1 amplitude × 100% for population spikes. PPR values were compared across experimental groups at each predefined interstimulus interval.

### 2.7. Chemicals

CaCl_2_ (#SLCD6507) was obtained from Sigma Aldrich, St. Louis, MO, USA. NaCl (#194848) and KCl (#194844) from MP Biomedicals, Irvine, CA, USA; MgCl_2_ (#63020) from Honeywell, Charlotte, NC, USA. Na_2_HPO_4_ (#A906159) was obtained from Merck, Darmstadt, Germany. NaHCO_3_ (#6885.2) was obtained from Carl Roth GmbH+Co.KG, Karlsruhe, Germany. Caffeine (#2071) was obtained from Calbiochem, Burlington, MA, USA. D-glucose (#Am-O188-0.5) was obtained from Helicon (Moscow, Russia). All reagents were of ultra-pure grade.

### 2.8. Statistics

Statistical analysis and graph preparation were performed using GraphPad Prism 10.4.0 (GraphPad Software, Boston, MA, USA) and Microsoft Excel 2016 (Microsoft, Redmond, WA, USA).

Normality was assessed using the Shapiro–Wilk and Kolmogorov–Smirnov tests. Because endpoint datasets and interval-specific paired-pulse datasets did not consistently meet the assumptions for normal distribution, these comparisons were performed using non-parametric statistics. TBS-induced long-term responses were analyzed using the Kruskal–Wallis test followed by the two-stage Benjamini, Krieger, and Yekutieli correction for multiple comparisons.

Input–output relationships were analyzed using a mixed-effects model with restricted maximum likelihood estimation (REML). The experimental group was treated as a between-slice factor, and relative stimulation intensity was treated as a within-slice repeated factor. Multiple comparisons were corrected using the two-stage Benjamini, Krieger, and Yekutieli procedure.

Paired-pulse responses were analyzed independently at each predefined interstimulus interval. Interstimulus interval was treated as a discrete experimental condition, and no common continuous response function across intervals was assumed for statistical inference. For each interstimulus interval, group comparisons were performed using the Kruskal–Wallis test followed by the two-stage Benjamini, Krieger, and Yekutieli correction for multiple comparisons.

Adjusted q values were used for multiple-comparison analyses, and differences were considered statistically significant at q < 0.05. The experimental unit was one animal represented by one hippocampal slice/recording. A formal a priori sample size calculation was not performed. Sample sizes were based on previous extracellular hippocampal slice electrophysiology studies using comparable protocols and on the number of stable recordings satisfying the inclusion criteria. Data are presented as mean ± standard error of the mean (SEM).

## 3. Results

### 3.1. Caffeine Attenuates TBS10-Induced Long-Term fEPSP Potentiation but Does Not Suppress TBS3-Induced E-S Potentiation

We first compared the effects of 30 μM caffeine on two TBS-induced CA1 responses. TBS10 was used to induce long-term fEPSP potentiation, whereas TBS3 was used to induce E–S potentiation, defined here as a sustained increase in population spike amplitude without a comparable increase in fEPSP slope.

**Figure 1:**
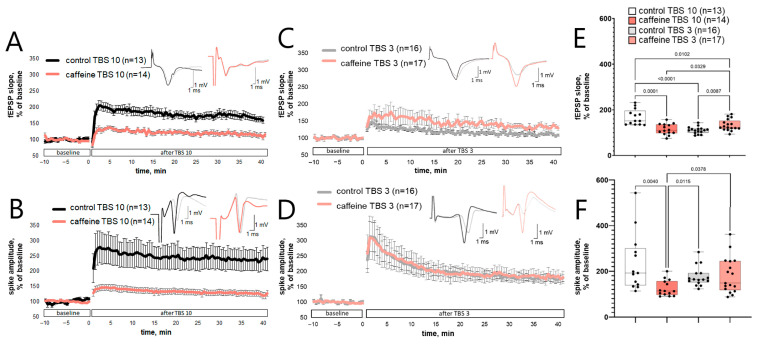
Caffeine attenuates TBS10-induced long-term fEPSP potentiation but does not suppress TBS3-induced E-S potentiation. (**A**–**D**) Time course of fEPSP slope (**A**,**C**) and population spike amplitude (**B**,**D**) before and after TBS10 (**A**,**B**) or TBS3 (**C**,**D**) in control and caffeine-treated slices. Data are shown as mean ± SEM. The x-axis shows time relative to the TBS application. Representative field responses are shown in the upper right of each panel; each trace represents the average of 10 consecutive recordings. Baseline responses are shown in gray, and post-TBS responses are shown in color. (**E**,**F**) Values averaged over 35–40 min after TBS for fEPSP slope (**E**) and population spike amplitude (**F**). Box plots show the median and interquartile range; whiskers indicate the range, and individual points represent individual slices. Statistical analysis was performed using the Kruskal–Wallis test followed by Benjamini, Krieger, and Yekutieli correction for multiple comparisons. For fEPSP slope: Kruskal–Wallis statistic = 25.16, *p* < 0.0001. For population spike amplitude: Kruskal–Wallis statistic = 12.57, *p* = 0.0057. Exact q values are shown in the figure. Group designations are defined in Section 2.

In control slices, TBS10 induced a persistent increase in both fEPSP slope and population spike amplitude. At 35–40 min after TBS10, the fEPSP slope reached 168 ± 34% of baseline, and population spike amplitude reached 237 ± 127% of baseline (n = 13; Figure 1A,B,E,F). In caffeine-treated slices, the corresponding values after TBS10 were lower: fEPSP slope reached 115 ± 24% of baseline, and population spike amplitude reached 125 ± 35% of baseline (n = 14; Figure 1A,B,E,F). Statistical comparison confirmed lower post-TBS10 values in caffeine-treated slices than in control slices for both fEPSP slope (q = 0.0001) and population spike amplitude (q = 0.004).

The response pattern after TBS3 differed from that observed after TBS10. In control slices, TBS3 did not produce a comparable increase in fEPSP slope, which reached 111 ± 15% of baseline at 35–40 min after stimulation, whereas population spike amplitude increased to 177 ± 43% of baseline (n = 16; Figure 1C–F). In caffeine-treated slices, fEPSP slope after TBS3 reached 133 ± 25% of baseline, and population spike amplitude reached 182 ± 79% of baseline (n = 17; Figure 1C–F). fEPSP slope was higher in the caffeine TBS3 group than in the control TBS3 group (q = 0.0087), although it remained lower than in the control TBS10 group (q = 0.0102). Population spike amplitude after TBS3 did not differ significantly between control and caffeine-treated slices.

Thus, 30 μM caffeine attenuated TBS10-induced long-term fEPSP potentiation and the accompanying increase in population spike amplitude, but did not suppress TBS3-induced E-S potentiation.

### 3.2. Caffeine Differentially Modifies CA1 Input–Output Relationships After TBS10 and TBS3

To determine whether the different effects of caffeine on TBS10- and TBS3-induced plasticity were associated with changes in CA1 excitability-related input–output relationships, we analyzed fEPSP slope and population spike amplitude across increasing stimulation intensities in slices without TBS and after TBS10 or TBS3.

**Figure 2:**
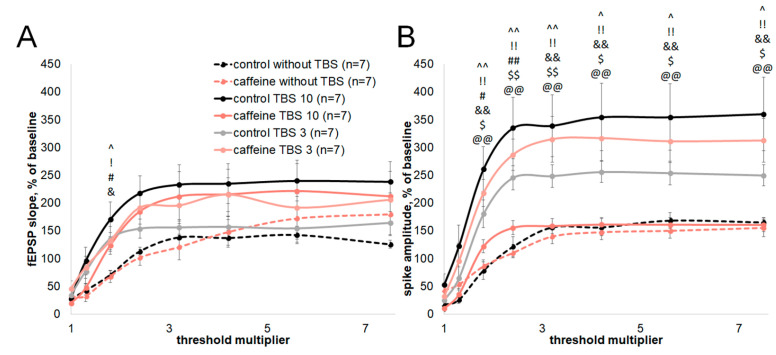
Caffeine differentially modifies CA1 input–output relationships after TBS10 and TBS3. Input–output curves for fEPSP slope (**A**) and population spike amplitude (**B**) in control and caffeine-treated slices without TBS, after TBS10, and after TBS3. Data are shown as mean ± SEM. The x-axis shows the threshold multiplier; the y-axis shows response magnitude expressed as a percentage of baseline. Input–output curves were analyzed separately for fEPSP slope and population spike amplitude using a mixed-effects model with restricted maximum likelihood estimation (REML), with stimulation intensity as the repeated factor. Multiple comparisons were corrected using the two-stage Benjamini, Krieger, and Yekutieli procedure. Significant comparisons are indicated as follows: ^ q < 0.05 and ^^ q < 0.01 for control without TBS vs. control TBS10; ! q < 0.05 and !! q < 0.01 for control without TBS vs. control TBS3; # q < 0.05 and ## q < 0.01 for caffeine without TBS vs. caffeine TBS10; & q < 0.05 and && q < 0.01 for caffeine without TBS vs. caffeine TBS3; $ q < 0.05 and $$ q < 0.01 for control TBS10 vs. caffeine TBS10; @@ q < 0.01 for caffeine TBS10 vs. caffeine TBS3. Group designations are shown in the figure legend and defined in Section 2.

For the fEPSP slope, the input–output changes were limited. Mixed-effects analysis detected significant TBS-related differences only at 1.8× threshold intensity. At this stimulation intensity, the fEPSP slope was increased after both TBS10 and TBS3 relative to non-tetanized groups, both under control conditions and during caffeine exposure (Figure 2A). No sustained separation between TBS10 and TBS3 was detected for the fEPSP slope across the stimulation range.

For population spike amplitude, the pattern was more clearly protocol-dependent. Under control conditions, both TBS10 and TBS3 increased population spike amplitude relative to slices without TBS over a broad range of stimulation intensities (1.8–7.5×; Figure 2B). During caffeine exposure, the TBS10-related increase was restricted to 1.8× and 2.4× threshold intensity, whereas after TBS3, population spike amplitude remained increased over the 1.8–7.5× range (Figure 2B).

Direct comparisons further showed that population spike amplitude was lower in caffeine-treated slices than in control slices after TBS10 over the 1.8–7.5× range. No corresponding reduction was detected after TBS3. Within caffeine-treated slices, population spike amplitude after TBS3 was higher than after TBS10 over the same 1.8–7.5× range.

Thus, caffeine differentially affected CA1 input–output relationships after TBS10 and TBS3. For the fEPSP slope, TBS-related changes were detected only at a single stimulation intensity. For population spike amplitude, caffeine attenuated and restricted the TBS10-related increase, whereas the TBS3-related increase was preserved across a broad stimulation range.

### 3.3. Caffeine Reduces Paired-Pulse Changes in Population Spike Responses

We next examined whether 30 μM caffeine affected paired-pulse responses and whether this effect was modified after TBS10- or TBS3-induced plasticity. The paired-pulse ratio (PPR) was calculated separately for fEPSP slope and population spike amplitude in slices without TBS and after TBS10 or TBS3.

**Figure 3:**
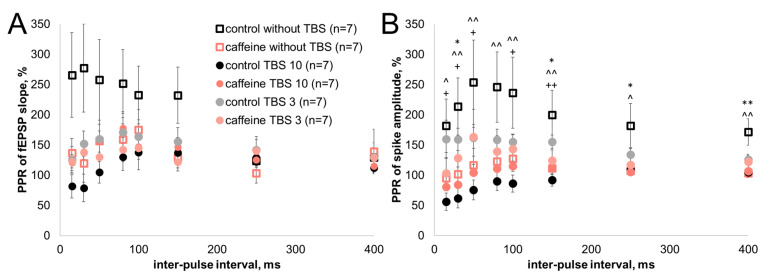
Caffeine reduces paired-pulse changes in population spike responses. Paired-pulse ratio (PPR) values for fEPSP slope (**A**) and population spike amplitude (**B**) in control and caffeine-treated slices without TBS and after TBS10 or TBS3. Data are shown as mean ± SEM. The x-axis shows the interstimulus interval, and the y-axis shows the PPR value. Statistical analysis was performed separately for each interstimulus interval using the Kruskal–Wallis test followed by Benjamini, Krieger, and Yekutieli correction for multiple comparisons. Significant comparisons are indicated as follows: * q < 0.05 and ** q < 0.01 for control without TBS vs. caffeine without TBS; ^ q < 0.05 and ^^ q < 0.01 for control without TBS vs. control TBS10; + q < 0.05 and ++ q < 0.01 for control TBS10 vs. control TBS3. Group designations are shown in the figure legend and defined in Section 2.

In control slices, PPR changes depended on the measured response component and on the preceding TBS protocol. For the fEPSP slope, TBS10 and TBS3 did not produce statistically significant changes in PPR relative to the control group without TBS (Figure 3A). For population spike amplitude, the control group without TBS showed PPR values above 100% at several interstimulus intervals, whereas after TBS10, PPR values were reduced and shifted below 100% at interstimulus intervals up to 150 ms (Figure 3B). After TBS3, population spikes, PPR values remained closer to those observed in the control group without TBS. Thus, under control conditions, preceding TBS10 changed paired-pulse population spike responses from facilitation toward depression, whereas this shift was not observed after TBS3.

Under caffeine exposure, PPR values remained close to 100% for both fEPSP slope and population spike amplitude in the group without TBS (Figure 3A,B). A similar pattern was observed after TBS10 and TBS3: no statistically significant differences were detected among the caffeine without TBS, caffeine TBS10, and caffeine TBS3 groups for either fEPSP slope or population spike amplitude. In the caffeine TBS3 group, population spike PPR values varied around 100%, with no consistent shift above or below this level.

Thus, 30 μM caffeine reduced paired-pulse deviations from 100%, most clearly for population spike amplitude. In control slices, these deviations included facilitation in the non-tetanized condition and a TBS10-associated shift toward depression; under caffeine exposure, stable facilitation- or depression-like paired-pulse changes were not detected after either TBS10 or TBS3.

## 4. Discussion

### 4.1. Differential Caffeine Sensitivity of TBS10-Induced fEPSP Potentiation and TBS3-Induced E-S Potentiation

The main result of the present study is that 30 μM caffeine separated two levels of CA1 plasticity-related responses: TBS10-induced long-term fEPSP potentiation was attenuated, whereas TBS3-induced E-S potentiation was preserved. Changes in input–output relationships and paired-pulse population spike responses are considered below as additional evidence that caffeine-sensitive modulation affected CA1 output dynamics.

The 30 μM caffeine concentration places these experiments within the low-micromolar range relevant for hippocampal slice plasticity. Caffeine intake through drinking water at 1 g/L has been reported to produce caffeine levels of 32 ± 4 μM in plasma, 23 ± 3 μM in cerebrospinal fluid, and approximately 22 μM in hippocampal tissue; on this basis, approximately 30 μM was proposed as an appropriate concentration for probing caffeine effects in hippocampal slices [7]. At this concentration range, caffeine is generally considered mainly in terms of adenosine receptor antagonism, whereas phosphodiesterase inhibition, intracellular Ca^2+^ release, and GABA_A_ receptor blockade require substantially higher concentrations [1,7,8,35]. In the present work, caffeine is therefore best considered as a physiologically relevant tool for probing the integrated effect of caffeine-sensitive adenosinergic modulation on different CA1 plasticity-related readouts.

The attenuation of TBS10-induced long-term fEPSP potentiation places this readout among hippocampal LTP responses that are sensitive to low-micromolar caffeine. A comparable caffeine concentration reduced HFS-induced fEPSP LTP in Schaffer collateral-CA1 synapses [7]. Pharmacological and genetic studies indicate that caffeine-sensitive adenosinergic modulation cannot be reduced to a single inhibitory action of adenosine receptors. In mouse Schaffer collateral-CA1 synapses, receptor-selective experiments provided evidence for two functionally distinct components of caffeine-sensitive adenosinergic modulation: an A1R-dependent component mainly associated with tonic control of basal glutamatergic transmission and an A_2A_R-dependent component required for full LTP expression [8]. The A_1_R component is also relevant to theta-patterned stimulation: DPCPX increased LTP induced by five theta bursts but did not further increase the near-maximal LTP induced by ten bursts, indicating that A_1_R blockade can enhance potentiation produced by abbreviated theta-burst trains without raising the ceiling of the response [36]. In this framework, the present TBS10 data extend caffeine-sensitive attenuation of hippocampal fEPSP potentiation to theta-burst-induced plasticity and set up the key comparison with TBS3: reducing the duration of theta-burst stimulation changed the observed action of caffeine from attenuation of long-term fEPSP potentiation to preservation of E-S potentiation.

The TBS3 result is important because shortening the theta-burst train did not simply reduce the magnitude of a TBS10-like fEPSP potentiation. Under TBS3 conditions, caffeine did not suppress E-S potentiation; rather, the long-term response was expressed with a different balance between the fEPSP and population spike components. This distinction is critical because E-S potentiation reflects a change in the relationship between synaptic input and spike output, not only a change in the magnitude of the field synaptic response. This interpretation is consistent with earlier work showing that theta-like stimulation protocols can induce E-S potentiation in the CA1 region. In CA1, primed-burst and patterned stimulation can produce a leftward shift in the EPSP–spike relationship and increase firing probability in a subset of intracellularly recorded pyramidal neurons without increasing EPSP slope [37]. Thus, the comparison between TBS10 and TBS3 indicates that reducing the duration of high-frequency stimulation transformed the expression of the caffeine-modulated long-term response from attenuation of fEPSP potentiation to preservation of E-S potentiation with an altered relationship between the fEPSP and population spike components.

This altered balance between the fEPSP and population spike components is captured by E-S potentiation, which reflects a plastic change in the transformation of synaptic input into spike output. Operationally, E-S potentiation is expressed as a leftward shift in the relationship between population spike amplitude and EPSP or fEPSP slope, indicating greater population spike output for a given synaptic input [16,17,18]. This component can be distinguished from synaptic LTP because population spike enhancement may exceed that predicted from EPSP/fEPSP potentiation and, in several experimental settings, may occur without proportional potentiation of the synaptic response [16,17,18]. Later work extended this view by showing that EPSP–spike coupling in CA1 can undergo bidirectional and input-specific plasticity and may involve inhibitory control, intrinsic excitability, and changes in input–output function [19,38,39,40]. Consistent with this framework, recent studies showed that CA1 spike output can diverge from excitatory synaptic efficacy and that neuromodulatory activation can induce E-S potentiation without proportional fEPSP potentiation [21,22].

This output-level interpretation is important because caffeine can facilitate memory performance in healthy animals and humans, whereas its effect on hippocampal fEPSP potentiation may be inhibitory. In healthy rodents, 1–30 mg/kg caffeine administered after training improved inhibitory-avoidance retention, and 3–10 mg/kg caffeine administered before testing improved retrieval in the same task [41]. In the Morris water maze, 0.3–10 mg/kg caffeine administered after training improved 48 h memory retention, and 3–10 mg/kg caffeine administered before testing produced a smaller retrieval-related improvement [42]. In healthy human subjects, 200 mg caffeine administered after studying enhanced 24 h object-discrimination memory, 200 mg caffeine increased recall of presented word-list items, 65 mg caffeine improved serial recall and running-memory performance in extraverts, and approximately 180 mg caffeine improved explicit memory in young adults tested in the early morning, their non-optimal time of day [43,44,45,46]. In otherwise healthy aging mice, chronic caffeine intake at 1 mg/mL prevented age-associated decline of object-recognition memory [47]. These findings place the preserved TBS3-induced E-S potentiation in a functional context: caffeine-related improvement of memory performance may rely on plastic changes that are not fully represented by fEPSP potentiation alone. The synaptic plasticity and memory hypothesis provides a framework for testing this possibility, because it evaluates whether a defined activity-dependent change contributes to encoding and trace storage through criteria such as detectability after learning, mimicry, anterograde alteration and retrograde alteration [48,49]. Within this framework, TBS3-induced E-S potentiation is a testable candidate mechanism for caffeine-related memory facilitation, because it represents a preserved change in EPSP–spike coupling under conditions in which 30 μM caffeine attenuated TBS10-induced fEPSP potentiation. This hypothesis should be tested by combining caffeine-sensitive behavioral paradigms with in vivo or ex vivo measurements of EPSP–spike coupling.

### 4.2. Input–Output and Paired-Pulse Responses Support Caffeine-Sensitive Modulation of CA1 Population Spike Responses

The input–output experiments add an important constraint to the interpretation of the TBS10/TBS3 dissociation. The caffeine effect was not limited to the final magnitude of long-term fEPSP potentiation; it also changed how evoked CA1 responses were expressed at the level of population spike amplitude across the stimulation range. For the fEPSP slope, input–output changes were restricted to a narrow part of the curve and did not produce a sustained separation between TBS10 and TBS3. Therefore, this readout did not account for the protocol-dependent difference in caffeine action. In contrast, population spike input–output curves showed a clearer protocol-dependent pattern. Under caffeine exposure, the TBS10-related increase in population spike amplitude was attenuated and restricted to low-to-intermediate stimulation intensities, whereas the TBS3-related increase remained expressed across a broader stimulation range. Direct comparison within caffeine-treated slices further separated these two outcomes, with higher population spike amplitudes after TBS3 than after TBS10. Thus, the input–output data support the same conclusion as the E-S analysis: shortening theta-burst stimulation changed the expression of caffeine-sensitive modulation from attenuation of the TBS10-related response to preservation of the TBS3-related population spike component.

The paired-pulse data extend this conclusion to responses to paired stimulation. Under 30 μM caffeine, paired-pulse modulation was most clearly changed for population spike amplitude, while fEPSP-slope PPR remained statistically less resolved. This difference between the two readouts is informative rather than secondary. Population spike PPR reflects how the second synaptic response is converted into spike output, and, therefore, it is expected to be more sensitive to changes in EPSP–spike coupling and local output control than fEPSP-slope PPR alone. A similar logic was proposed by I.V. Kudryashova, who argued that PPR changes can be more clearly detected from population spike amplitude than from fEPSP slope because neuronal hyperpolarization has little effect on the magnitude of the input synaptic current [50]. In the same work, the reduction in short-term facilitation at short interstimulus intervals was associated with GABA receptor activation, whereas changes at longer intervals were considered to involve additional mechanisms [50].

This framework fits the present paired-pulse results: caffeine reduced the protocol-dependent deviations of population spike PPR from 100%. In control slices, paired-pulse population spike responses changed with the preceding stimulation history: non-tetanized slices showed PPR values above 100% at several intervals, whereas after TBS10 the response shifted toward lower PPR values, including values below 100% at short interstimulus intervals. Under caffeine exposure, population spike PPR remained closer to 100% across the analyzed conditions, and stable shifts above or below this level were not detected after either TBS10 or TBS3. Thus, the paired-pulse data indicate that caffeine reduced short-term changes in population spike responses to paired stimulation, rather than selectively affecting only one direction of PPR change.

### 4.3. Receptor-Selective Mechanisms and Future Experimental Directions

The present data define several mechanistic questions that should be addressed directly in future work. The comparison between TBS10 and TBS3 suggests that the direction of caffeine-sensitive modulation depends on the duration of theta-burst stimulation and on the electrophysiological readout used to evaluate the CA1 response. Receptor-selective experiments are therefore the next critical step. Selective A_1_R and A_2A_R ligands, adenosine deaminase, and receptor-specific models will be required to determine how A_1_R-dependent control of basal transmission, A_2A_R-dependent support of LTP, and their interaction during patterned activity contribute to the different caffeine sensitivity of TBS10-induced fEPSP potentiation and TBS3-induced E-S potentiation.

The cellular locus of this modulation also remains an important question. GABAergic transmission, NMDA receptor-dependent induction, AMPA receptor-dependent synaptic expression, and intrinsic excitability may each contribute to the balance between fEPSP and population spike components, but these mechanisms require direct testing with targeted pharmacology and intracellular or whole-cell measurements. Such experiments would clarify how caffeine shifts the expression of CA1 plasticity between synaptic and spike-output components after different theta-burst protocols. A separate but closely related step is to test the functional relevance of this dissociation by combining caffeine-sensitive behavioral paradigms with in vivo or ex vivo measurements of EPSP–spike coupling.

## 5. Conclusions

In this study, 30 μM caffeine differentially affected selected CA1 plasticity-related electrophysiological responses in mouse hippocampal slices. Caffeine attenuated TBS10-induced long-term fEPSP potentiation and the accompanying increase in population spike amplitude, but did not suppress TBS3-induced E-S potentiation. Input–output analysis further showed that caffeine attenuated and restricted the TBS10-related increase in population spike amplitude, whereas the TBS3-related increase remained expressed across a broader stimulation range. For the fEPSP slope, input–output changes were limited and did not produce a sustained separation between TBS10 and TBS3. Caffeine also reduced paired-pulse deviations from 100%, most clearly for population spike amplitude, and this pattern was preserved after both TBS10 and TBS3.

Together, these findings indicate that low-micromolar caffeine does not uniformly suppress CA1 plasticity-related responses. Instead, caffeine-sensitive modulation depends on the induction protocol and on the electrophysiological readout, with different effects on long-term fEPSP potentiation, EPSP–spike coupling, stimulation-dependent population spike amplitude, and paired-pulse population spike responses. The preservation of E-S potentiation under caffeine exposure identifies EPSP–spike coupling as an important candidate readout for future studies of caffeine-related memory effects. Receptor subtype-specific mechanisms and the functional relevance of this dissociation require direct testing using receptor-selective tools and behavioral paradigms combined with in vivo or ex vivo measures of EPSP–spike coupling.

## Data Availability

The raw data supporting the conclusions of this article will be made available by the authors on request.

## Abbreviations

The following abbreviations are used in this manuscript:

A_1_R	Adenosine A1 Receptor
A_2A_R	Adenosine A2A Receptor
ACSF	Artificial Cerebrospinal Fluid
AMPA	α-amino-3-hydroxy-5-methyl-4-isoxazolepropionic acid
AMPAR	AMPA Receptor
EPSP/f-EPSP	Excitatory Postsynaptic Potential (Field EPSP)
E-S potentiation	EPSP–spike-Potentiation
GABA	Gamma-Aminobutyric Acid
GABA_A_	GABA type A receptor
ISI	Interstimulus Interval
LTP	Long-Term Potentiation
NMDA	N-methyl-D-aspartate
NMDAR	NMDA Receptor
PPR	Paired-Pulse Ratio
PPS	Paired-Pulse Stimulation
TBS	Theta-Burst Stimulation
